# Recent Advances in Nanomechanical Membrane-Type Surface Stress Sensors towards Artificial Olfaction

**DOI:** 10.3390/bios12090762

**Published:** 2022-09-16

**Authors:** Kosuke Minami, Gaku Imamura, Ryo Tamura, Kota Shiba, Genki Yoshikawa

**Affiliations:** 1 Center for Functional Sensor & Actuator (CFSN), Research Center for Functional Materials, National Institute for Materials Science (NIMS), 1-1 Namiki, Tsukuba 305-0044, Ibaraki, Japan; 2 International Center for Materials Nanoarchitectonics (MANA), National Institute for Materials Science (NIMS), 1-1 Namiki, Tsukuba 305-0044, Ibaraki, Japan; 3 Graduate School of Information Science and Technology, Osaka University, 1-2 Yamadaoka, Suita 565-0871, Osaka, Japan; 4 Research and Services Division of Materials Data and Integrated System (MaDIS), National Institute for Materials Science, 1-1 Namiki, Tsukuba 305-0044, Ibaraki, Japan; 5 Graduate School of Frontier Sciences, The University of Tokyo, 5-1-5 Kashiwanoha, Kashiwa 277-8568, Chiba, Japan; 6 Materials Science and Engineering, Graduate School of Pure and Applied Science, University of Tsukuba, 1-1-1 Tennodai, Tsukuba 305-8571, Ibaraki, Japan

**Keywords:** Membrane-type Surface stress Sensor (MSS), nanomechanical sensors, static mode operation, artificial olfaction, machine learning

## Abstract

Nanomechanical sensors have gained significant attention as powerful tools for detecting, distinguishing, and identifying target analytes, especially odors that are composed of a complex mixture of gaseous molecules. Nanomechanical sensors and their arrays are a promising platform for artificial olfaction in combination with data processing technologies, including machine learning techniques. This paper reviews the background of nanomechanical sensors, especially conventional cantilever-type sensors. Then, we focus on one of the optimized structures for static mode operation, a nanomechanical Membrane-type Surface stress Sensor (MSS), and discuss recent advances in MSS and their applications towards artificial olfaction.

## 1. Introduction

**Figure 1:**
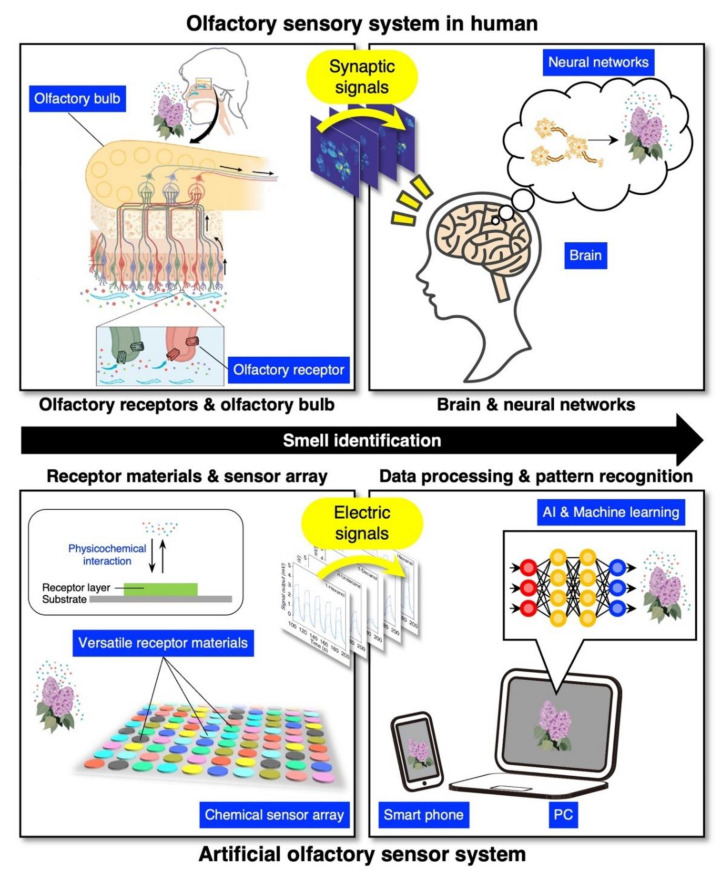
Schematic illustrations of olfactory sensory system in human and artificial olfaction system. Part of images are reprinted with permission from Ref. [22], Copyright 2007, European Molecular Biology Organization; and from Ref. [23], the authors licensed under CC-BY 4.0.

In nature, it is full of various odors and humans as well as other organisms tend to recognize their surroundings by odors [1]. Each odor is usually composed of dozens to thousands of different molecules out of more than 400,000 types of odorous/odorless molecules [2]. In most cases, we detect such a complex odor as a simultaneous interaction of various types of molecules with our olfactory receptors and recognize the odor by comprehensively analyzing the signals mediated by various receptors in the brain (Figure 1). In contrast to other senses that perceive physical stimuli (i.e., light for eye, sound for ear, and pressure for skin), olfactory sensors have not been practically commercialized because of such complexity of the sense of smell and lack of a comprehensive understanding of the chemical interactions between the receptors and the analytes.

The concept of an “artificial olfaction” was first proposed in 1982 by Persaud et al. as a model of a nose using an array of different types of sensors and resultant unique signal patterns to discriminate specific odors [3]. The system of the artificial olfaction is inspired by the olfactory perception pathway (Figure 1). Specifically, receptor materials coated on sensing elements correspond to olfactory receptors, sensing elements and transducers work as olfactory cells and bulbs, and pattern recognition analysis plays a role of neural activity in the brain. The constructed system is called an electronic nose (e-nose) [4,5]. The recent achievements in size reduction of sensing elements by nanotechnologies have accelerated olfactory sensor technologies. In the last decade, the olfactory sensor technology has evolved significantly along with the evolution of data processing technologies, including artificial intelligence (AI) and machine learning algorithms [6,7,8,9,10]. There are lots of studies applying current olfactory sensor technology to various fields, such as food, agricultural, environmental, medical, and healthcare fields [6,11,12,13,14,15,16,17,18]. Moreover, the olfactory sensor technology covers the detection of odors that cannot be sensed by humans, such as carbon monoxide [19] and hydrogen [20,21]. In such developments of the olfactory sensors, gas (chemical) sensors have gained significant attention as they play a critical role in detecting odors [11,14,15,16,17].

**Figure 2:**
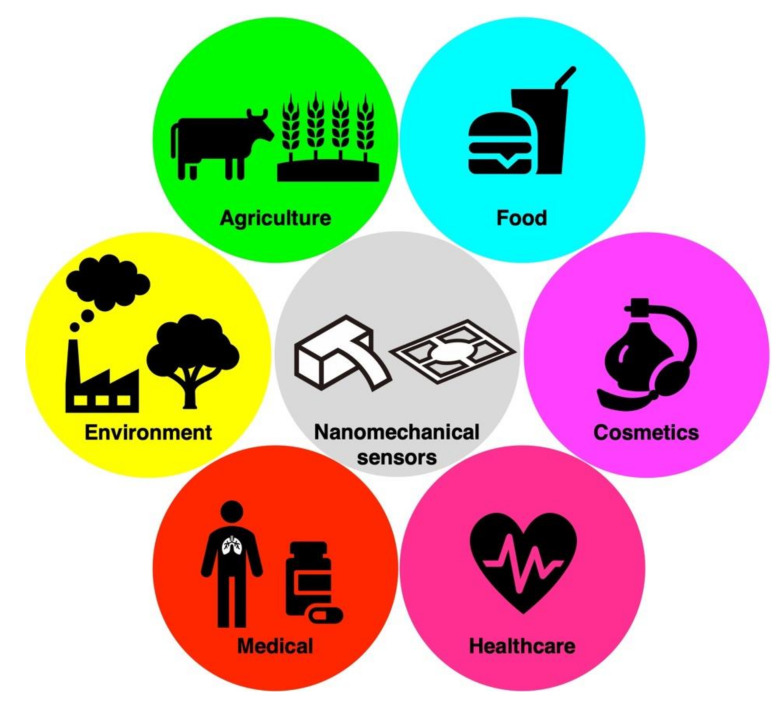
Possible applications of nanomechanical sensors.

Among a wide variety of chemical sensors, nanomechanical sensors have received significant attention, as they find plenty of applications in many different research fields (Figure 2) [11,24,25,26,27,28,29,30,31,32,33]. In human olfactory perception, it is known that there are approximately 400 different kinds of olfactory receptors [34], which express wide varieties of chemical selectivity, providing unique patterns of odors. To develop artificial olfaction, it is important to construct certain numbers of receptor materials, which have chemical selectivity, to apply multivariate analyses, including pattern recognition. In this context, nanomechanical sensors are one of the ideal sensing platforms for olfactory sensors because of its intrinsic versatility. According to their working principle, nanomechanical sensors detect volume- and/or mass-induced mechanical changes of a sensing element [32]. Since it has been observed that almost all kinds of solid materials, including organic small molecules, polymers, self-assembled nanomaterials, inorganic nanoparticles, and biomolecules, exhibit mechanical deformation upon gas sorption, various types of solid materials can be utilized as a receptor material, providing a wide range of chemical selectivity and sensitivity. Lang et al. reported the first applicability of nanomechanical sensor arrays to the artificial olfaction [35,36,37]. In this review, we aim to summarize the recent studies in the field of olfactory sensing by focusing on nanomechanical sensors. We first address the fundamental research on nanomechanical sensors, including their background theories. We then focus on a specific geometry of nanomechanical sensors with superior performance: a Membrane-type Surface stress Sensor (MSS) [38,39]. Finally, we summarize the recent advances of MSS and their applications as an artificial olfaction.

## 2. Nanomechanical Sensors

As we emphasized, nanomechanical sensors can provide a promising sensing platform for artificial olfaction. In 1994, Gimzewski et al. reported the first chemical sensing application using a nanomechanical microcantilever-type sensor [40]. They used the static bending of a cantilever to detect the catalytic reaction proceeding on the surface of the cantilever. In the same year, Thundat et al. demonstrated a mass detection with picogram resolution using the dynamic behavior of a nanomechanical cantilever-type sensor [41]. They focused on the cantilever resonance frequency shifts induced by the exposure of a metal-coated cantilever to humidity or mercury vapor. Then, many research groups have demonstrated that nanomechanical sensors can detect not only a variety of targets, such as moisture [41,42,43] and mercury vapor [41,44], but also various chemical/physical phenomena, including the formation of a self-assembled monolayer [45], DNA hybridization [46,47,48], a single spin [49,50], and quantum state [51,52]. Regarding the working principle of nanomechanical sensors whose flexible structures deform at the nanoscale, they are following two major types of operation modes: the static mode and dynamic mode [32,53,54,55,56,57,58]. In this section, we will briefly review these two operation modes. Then, we focus on the static mode operation with their theoretical models for the effects of surface stress as well as the effects of the sorption of analytes. At the end of this section, among various types of nanomechanical sensors [59,60,61], we will introduce a specific geometry of nanomechanical sensors with electrical readout having improved sensitivity: MSS [38].

### 2.1. Static and Dynamic Mode

**Figure 3:**
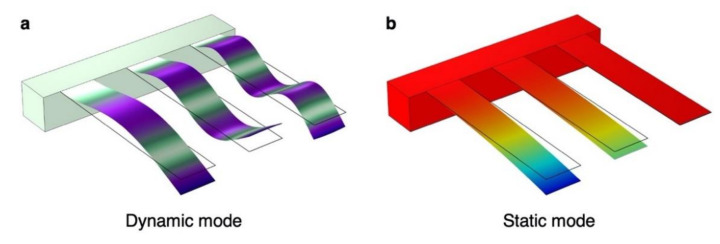
Schematic illustrations of two different operation modes of cantilever-type nanomechanical sensors simulated by finite element analysis (FEA) through COMSOL Multiphysics. (**a**) Dynamic mode operation, in which a nanomechanical sensor detects sorption-induced changes in the resonance frequencies with mass effect, stiffness effect, and effect of surface stress. (**b**) Static mode operation, in which a nanomechanical sensor detects changes in the deflection caused by the sorption-induced surface stress. It is important to note that the bending of a cantilever plate is not caused by the gravity effect.

One of the typical geometries of nanomechanical sensors is a cantilever. The cantilever-type sensors can detect two physical parameters: volume and/or mass of target molecules. To measure the volume and mass of target molecules, there are two different operation modes as mentioned above: dynamic and static modes (Figure 3) [32,53,54,55,56,57,58]. While the static mode detects changes in the deformation state of a nanomechanical sensor, the dynamic mode detects changes in the mechanical resonances.

The concept of dynamic mode operation is the same with those as it is for various resonators, such as quartz crystal microbalance (QCM). In this mode, the shift in resonance frequency is measured. This shift is due to the changes in effective mass induced by the adsorption of analytes on a cantilever. Since signals can be directly correlated with the basic property of adsorbates, i.e., mass, the dynamic mode is a useful and powerful technique to derive quantitative information. As the sensitivity generally depends on the resonance frequency determined by the size of a cantilever, a nanometer-scale cantilever operates at very high frequency bands (ca. 30–300 MHz) and marks several milestones, such as ca. 7 zeptogram (10^−21^ g) resolution by a cryogenically cooled apparatus in an ultrahigh vacuum (below 10^−10^ torr) [62], and the mass resolution being less than 1 attogram (10^−18^ g) in the air at room temperature [63]. Because of its high sensitivity, nanomechanical sensors in dynamic mode can be utilized for a new type of mass spectrometry known as nanomechanical mass spectrometry [26,28,61,64,65,66,67,68,69]. To improve the sensitivity further for the dynamic mode operation, various studies have reported, such as the use of other functional structures [32,43,70,71,72].

In contrast to the dynamic mode operation, the static mode is known as one of the representative operation modes of cantilever-type nanomechanical sensors. It measures surface stress, which is generally not easy to be measured with other sensing techniques. One of the major advantages of the static mode is that a cantilever does not suffer from damping because the bending motion caused by the sorption-induced surface stress is slow enough, minimizing the damping in most cases. Moreover, the static mode operation does not require an actuator for mechanical vibration, enabling the miniaturization of the entire measurement system.

### 2.2. Effects of Surface Stress in Static Mode

**Figure 4:**
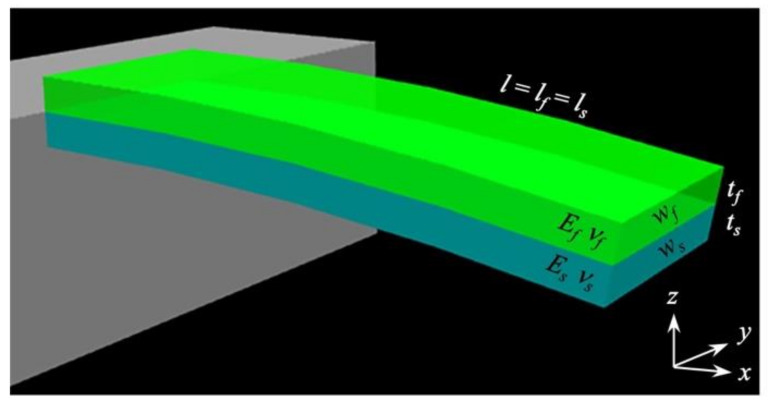
Schematic illustration of conventional cantilever-type nanomechanical sensors in the Cartesian coordinates. Reprinted with permission from Ref. [75]. Copyrights 2012, Elsevier.

Theories and models for the static mode operation date far back to 1909, when Stoney published his equation to relate the surface stress $\sigma_{\mathrm{surf}}$ with the bending curvature κ of a free-standing plate [73], which is known as Stoney’s equation. Stoney derived the curvature–stress relation using plate theory composed of stress bearing a thin film of thickness $t_{f}$ deposited on a substrate of thickness $t_{s}$ as follows:

(1) $$\kappa =\frac{6\left(1-\nu_{s}\right)t_{f}}{E_{s}t_{s}^{2}}\sigma_{f},$$

where $E_{s}$ and $\nu_{s}$ are Young’s modulus and Poisson’s ratio of a substrate of a cantilever, respectively (Figure 4). The Stoney’s equation is widely utilized to estimate the deflection of a cantilever beam. According to the Stoney’s equation with the relations of constant curvature $\kappa =2\Delta \Delta z/l_{s}^{2}$ [73,74], where $l_{s}$ is the length of the cantilever plate, as well as the conversion of three-dimensional internal stress $\sigma_{f}$ to two-dimensional surface stress $\sigma_{\mathrm{surf}}$ as

(2) $$\sigma_{\mathrm{surf}}=\sigma_{f}t_{f},$$

the deflection of a free end of a cantilever Δz caused by surface stress can be rewritten as

(3) $$\Delta z=\frac{3\left(1-\nu_{s}\right)l_{s}^{2}}{E_{s}t_{s}^{2}}\sigma_{\mathrm{surf}}.$$

Although Equation (3) has been widely utilized in various fields not only for nanomechanical sensors but also for the fabrication of micro electromechanical systems (MEMS) to estimate the internal stress by the curvature of a silicon wafer, the Stoney’s equation does not account for the clamping effect of a cantilever, resulting in the loss of accuracy to describe the curvature, especially near the clamping region.

To estimate surface stress from the bending of the cantilever plate, the relation between the bending and the surface stress has been proposed. The differential equation governing the bending of a rectangular plate is the so-called biharmonic equation, which is given by $\nabla^{4}w=0$ in Cartesian coordinates, where w is the out-of-plane displacement of the plate along the *z* coordinate direction. This analytical solution with cantilever boundary conditions was attempted by Zeng et al. [76]. They proposed a solution in terms of Fourier cosine series. By imposing the appropriate boundary conditions, a simultaneous linear equation can be obtained with the coefficients of the cosine series as unknown. However, to calculate their coefficients, it is necessary to solve unconditional infinite equations with slow convergence, suggesting that the boundary conditions of the clamped and free end of the plate are inconsistent at the common corners. Sader derived approximated solutions to the problem in the asymptotic limits of high and very low aspect ratios. In the case of a very low aspect ratio (i.e., L≫b), the solution is the following equation as [77]

(4) $$w\left(x,y\right)=\frac{\kappa }{2}\left[\left(x^{2}+y^{2}\right)+f\left(x\right)+y^{2}g\left(x\right)\right],$$

where the functions $f\left(x\right)$ and $g\left(x\right)$ are given by

(5) $$f\left(x\right)=-b^{2}\left\{\frac{1}{12}+2\nu_{s}\left[\frac{1}{\tau_{1}^{2}}+\frac{1}{\tau_{2}^{2}}+\frac{1}{\tau_{1}\tau_{2}}-\left(\frac{1}{\tau_{1}}+\frac{1}{\tau_{2}}\right)\frac{x}{b}\right]-\overset{2}{\underset{i=1}{\sum }}d_{i}\left(\frac{1}{12}+\frac{2\nu_{s}}{\tau_{i}^{2}}\right)e^{-\tau_{i}\frac{x}{b}}\right\},\;g\left(x\right)=-\overset{2}{\underset{i=1}{\sum }}d_{i}e^{-\tau_{i}\frac{x}{b}},$$

with the coefficients $d_{i}$ and $\tau_{i}$ as

(6) $$d_{i}=\frac{\tau_{3-i}}{\tau_{3-i}-\tau_{i}},$$

(7) $$\tau_{i}=2\sqrt{3}{\left\{5\left(1-\nu_{s}\right)+{\left(-1\right)}^{i}{\left[10\left(1-\nu_{s}\right)\left(2-3\nu_{s}\right)\right]}^{\frac{1}{2}}\right\}}^{\frac{1}{2}},$$

where L and b denote the length and width of a rectangular plate. Note that the constant curvature κ is given by [77]

(8) $$\kappa =\frac{6\left(1-\nu_{s}\right)}{E_{s}t_{s}^{2}}\sigma_{\mathrm{surf}}.$$

In the case of long aspect ratio L≫b, the analytical solution states that the curvature of the cantilever follows the Stoney’s equation in Equation (3) far from the clamped edge and then decays exponentially with a characteristic length of the order of b. A different approach was also reported by Tamayo et al. for a cantilever with a relatively small aspect ratio with L>b [78]. They obtained a simple formula for the averaged transversal and longitudinal curvatures with Poisson’s ratio-dependent coefficient.

**Figure 5:**
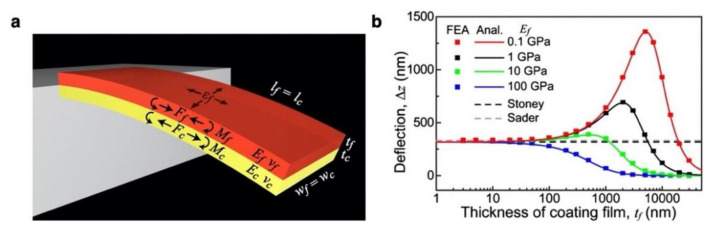
An analytical solution of cantilever-type nanomechanical sensor. (**a**) Schematic illustration of a cantilever coated with a film. (**b**) Dependence of a cantilever deflection on the thickness of coating films $t_{f}$ with various Young’s moduli of coating films $E_{f}$ ranging from 0.1 to 100 GPa calculated by Equation (9). The values calculated by FEA are represented with solid squares. Black and gray dashed lines correspond to the cantilever deflection calculated by the Stoney’s equation in Equation (3) and Sader’s model in Equation (4) with Equation (8), respectively. $l_{f}=l_{s}=$ 500 [µm]; $w_{f}=w_{s}=$ 100 [µm]; $t_{s}=$ 1 [µm]; $E_{s}=$ 170 [GPa]; $\nu_{s}=$ 0.28; $\nu_{f}=$ 0.30; and $\sigma_{\mathrm{surf}}=$ 0.1 [N m^−1^]. Reprinted with permission from Ref. [80]. Copyrights 2012, American Institute of Physics.

To consider the effect of the property of a receptor layer, the Timoshenko beam theory, which was originally developed to analyze a bimetal strip, can be used [79]. The Timoshenko beam theory includes all relevant physical properties of both the cantilever substrate and coating film. On the basis of the Timoshenko beam theory, an analytical model for the static deflection of a nanomechanical cantilever-type sensor coated with a solid layer, was derived by Yoshikawa [80]. A simple cantilever covered by a coating film, in which isotropic internal strain $\varepsilon_{f}$ is applied, is assumed as shown in Figure 5a. The deflection of the cantilever Δz is described as [80]

(9) $$\Delta z=\frac{3\left(t_{s}+t_{f}\right)l_{s}^{2}}{\left(A+4\right)t_{f}^{2}+\left(A^{-1}+4\right)t_{s}^{2}+6t_{f}t_{s}}\varepsilon_{f},$$

with

(10) $$A=\frac{E_{s}w_{s}l_{s}}{1-\nu_{s}}/\frac{E_{f}w_{f}l_{f}}{1-\nu_{f}},$$

where $E_{f}$ and $\nu_{f}$ are the Young’s modulus and Poisson’s ratio of a coating film, respectively; $w_{s}$ and $w_{f}$ correspond to the widths of a cantilever and a coating film, respectively. By substituting the relation between the internal strain $\varepsilon_{f}$ and the internal stress $\sigma_{f}$, which is given by

(11) $$\varepsilon_{f}=\frac{E_{f}}{1-\nu_{f}}\sigma_{f},$$

and Equation (2) into Equation (9), the deflection of a cantilever Δz can be described as a function of surface stress $\sigma_{\mathrm{surf}}$. In the case of $t_{s}\gg t_{f}$, Equation (9) reduces to the Stoney’s equation in Equation (3). As clearly seen in Figure 5b, the derived equation well expresses the dependence of the thickness and the coating film properties, while the Stoney’s equation only covers the case of $t_{s}\gg t_{f}$.

Various analytical solutions for understanding the relations between the bending and the surface stress [81,82,83] as well as the extended Stoney’s equations with complex systems [84,85,86,87,88] have been proposed; however, these models are still limited to simple and specific models. Alternatively, finite element analyses (FEA) can numerically simulate large varieties of complicated systems, including various cantilever-type nanomechanical sensors and any other type of nanomechanical sensors [38,39,75,77,78,80,81,89,90,91,92], providing some guidelines of the effect of the surface stress for the static mode nanomechanical sensing.

### 2.3. Sorption Kinetics and Viscoelastic Behaviors of Receptor Materials in Static Mode

Signal response in the static mode operation of nanomechanical sensing is derived from the surface stresses exerted on the surface of a substrate. This surface stress is induced by the sorption of the analyte on the coating film. While the effect of the surface stresses has been well investigated, as described above, the difference in signal response for each analyte is governed by the physicochemical interaction between an analyte and a coating film. Therefore, an understanding of the relationship between such physicochemical interactions and the sorption-induced stress/strain is important in identifying the analyte as practical applications for artificial olfaction. Here, we present related theoretical studies of physicochemical interactions in nanomechanical sensing based on sorption kinetics.

In the case of the sorption-induced nanomechanical sensing, there are several investigations using nanomechanical cantilever-type sensors [93,94,95]. In the models, the sorption-induced internal strain in a coating film $\varepsilon_{f}$ is approximated as follows [95]:

(12) $$\varepsilon_{f}=\sqrt[{3}]{1+Cv_{a}}-1,$$

which has the linear approximation given by

(13) $$\varepsilon_{f}=\frac{1}{3}Cv_{a},$$

for small volume expansion (i.e., $\varepsilon_{f}\ll 1$), where C is the concentration of absorbed analyte in the coating film; $v_{a}$ is the specific volume of the absorbed analyte. The absorption-induced strain can, therefore, be assumed to be directly proportional to the concentration of absorbed analyte in the coating film C. From Equation (9) with Equation (13), the deflection at the free end of the cantilever Δz can be approximated to be directly proportional to the concentration of an analyte in the coating film.

The absorption process of an analyte into the bulk of a coating is generally rate limited by the diffusion of the analyte across the surface barrier and into a coating film [93]. If the diffusion is Fickian, then the rate of absorption will be proportional to the difference between the equilibrium concentration in the coating film $K_{p}C_{g}\left(t\right)$ and the concentration already absorbed analyte into the coating $C\left(t\right)$ as [93,95,96]

(14) $$\frac{d}{dt}C\left(t\right)=\frac{1}{\tau_{s}}\left[K_{p}C_{g}\left(t\right)-C\left(t\right)\right],$$

where $K_{p}$ is the partition coefficient and $C_{g}\left(t\right)$ is the concentration of the analyte in the gas phase; $\tau_{s}$ or $1/\tau_{s}$ is the diffusion time constant or a single decay rate, respectively.

In the case of gas sensing using nanomechanical sensors, a gas line introducing sample gas by carrier gas and a purge gas line to desorb the sample gas molecules absorbed in the coating film are switched. Since the sample gas is generally introduced by the continuous flow of headspace gas or vapors generated by bubbling liquid samples, the concentration of analyte in sample gas can be assumed to be homogeneous in time (i.e., $C_{g}\left(t\right)=C_{g}$). Thus, a rectangular injection of analyte with a constant rate can be considered (see also Figure 3a in Ref. [96] and Equation (10) in Ref. [96]). The differential equation in Equation (14) can be solved as a step function:

(15) $$C\left(t\right)=\left\{\begin{array}{ll} 0, & t<t_{0}\\ K_{p}C_{g}\left(1-e^{-\frac{t-t_{0}}{\tau_{s}}}\right), & t_{0}\leq t<t_{1}\\ K_{p}C_{g}\left(1-e^{-\frac{t_{1}-t_{0}}{\tau_{s}}}\right)e^{-\frac{t-t_{1}}{\tau_{s}}}, & t_{1}\leq t \end{array}\right.,$$

where $t_{0}$ and $t_{1}$ are the times when sampling and purging starts, respectively (see also Figure 3 in Ref. [32]).

**Figure 6:**
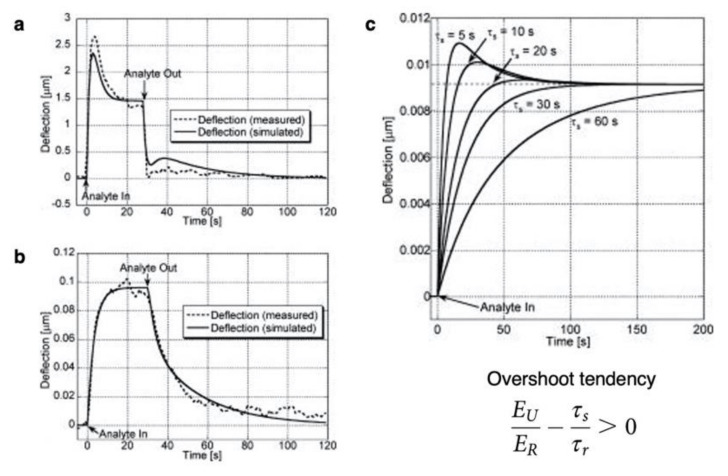
Comparison of experimentally measured signal responses of cantilever-type nanomechanical sensors and calculated bending responses based on Wenzel’s model. (**a**,**b**) Comparison of experimentally measured signal responses for (**a**) a cyclodextrin-coated cantilever exposed to trichloroethylene and (**b**) a poly(diphenoxyphosphazene)-coated cantilever exposed to di-isopropylmethylphsphonate. (**c**) Typical calculated bending response for a cantilever-type nanomechanical sensor during absorption of analytes for various sorption times but same steady-state sorption-induced elongation. Reprinted with permission from Ref. [95]. Copyright 2008, American Institute of Physics.

The analytical solution of the absorption process can be expressed as a typical first-order response; however, most of the signal responses from the nanomechanical sensing do not follow the above derived equation because a large number of receptor materials exhibit viscoelastic behavior. The viscoelastic properties arise from dynamic differences on molecular rearrangements [97]. To overcome this problem, Wenzel et al. proposed a theoretical model for a cantilever-type nanomechanical sensor coated with a viscoelastic material [95]. The theoretical models are derived from the simplest three-parameter solid model [95,98]:

(16) $$\tau_{r}E_{U}\frac{d}{dt}\varepsilon \left(t\right)+E_{R}\varepsilon \left(t\right)=\tau_{r}\frac{d}{dt}\sigma \left(t\right)+\sigma \left(t\right),$$

where $E_{U}$ and $E_{R}$ denote the unrelaxed (instantaneous) and relaxed (asymptotic) moduli, respectively, and $\tau_{r}$ is the time constant of stress relaxation. The three-parameter solid model describes the stress/strain relationship in a viscoelastic solid that exhibits both viscous and elastic properties. As proposed by Wenzel et al. [95], the derived general differential equations from Equation (16) can be greatly simplified as

(17) $$\frac{d}{dt}\sigma \left(t\right)=-\frac{\sigma \left(t\right)}{\tau_{r}}+\frac{E_{R}\lambda }{\tau_{r}}\left(\frac{E_{U}}{E_{R}}-\frac{\tau_{s}}{\tau_{r}}\right)C\left(t\right)-\frac{E_{U}K_{p}\lambda }{\tau_{s}}C_{g}\left(t\right),$$

when the coating film is significantly soft (i.e., $E_{f}\ll E_{s}$) or thin (i.e., $h_{f}\ll h_{s}$), where $\lambda =\frac{1}{3}v_{a}$. By substituting Equation (15) into Equation (17), the general differential equation that governs the stress with a rectangular injection can be solved. As presented in Figure 6, the derived equation clearly fits well with viscoelastic polymer-coated signals that respond upon exposure to an analyte. Importantly, nanomechanical sensing signals often exhibit overshoot trends in the injection process and undershoot trends in the purge process (Figure 6b). The derived equation based on Wenzel’s model clearly simulates these overshoot/undershoot trends. The derived condition for which the response exhibits the overshoot/undershoot trends is

(18) $$\frac{E_{U}}{E_{R}}-\frac{\tau_{s}}{\tau_{r}}>0,$$

only if $E_{U}>E_{R}$ with a long enough duration, as shown in Figure 6c.

**Figure 7:**
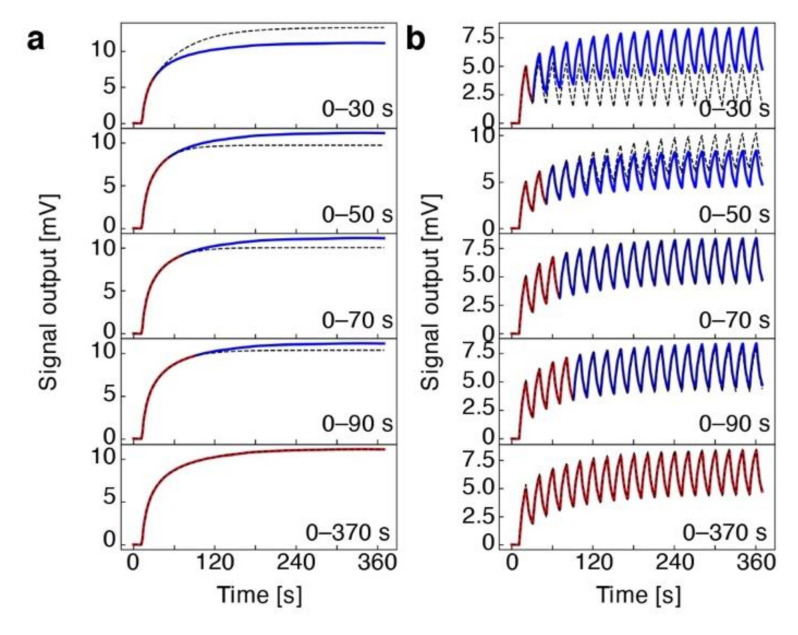
Fitting accuracy between a single injection signal response and multistep injection–purge cycles. (**a**,**b**) Comparison of experimentally measured signal responses of polycaprolactone-coated MSS exposed to 1,2-Dichlorobenzeneat the concentration of $P_{a}/P_{o}=$ 30% and calculated signal responses based on a single injection signal response model (**a**) and a multistep injection–purge cycles model (**b**). Red colored signal responses are used for optimizing each fitting curve. Black dashed lines are the corresponding fitting curves. Reprinted from Ref. [96], the authors licensed under CC-BY 4.0.

It should be noted that the derived equation based on Wenzel’s model can be utilized for extracting fitting parameters by using a signal response, which reaches the steady state or equilibrium state. However, when the measured signal response does not reach the steady state, the parameters extracted from the experimental results cannot predict the entire signal responses (Figure 7a). Recently, Minami et al. extended the analytical solution based on Wenzel’s model to the multistep injection–purge cycle system, which can be effectively utilized to predict and/or analyze the signal responses of a nanomechanical sensor without measuring the signal until it reaches the steady state [96]. In nanomechanical sensing, the multistep injection–purge cycles are often used to obtain repetitive signal patterns (Figure 7b). For the derivation of the multistep injection–purge cycles, they considered a rectangular pulse wave-like sequence, in which the concentration of an analyte in the gas phase $C_{g}\left(t\right)$ can be described as a step function:

(19) $$C_{g}\left(t\right)=\left\{\begin{array}{ll} 0, & t<t_{0}\\ C_{g}, & t_{2\left(n-1\right)}\leq t<t_{2n-1}\\ 0, & t_{2n-1}\leq t<t_{2n} \end{array}\;\;\;\;\;\;\left(n=1,2,\ldots \right)\;\right.,$$

where *n* indicates the number of the *n*-th injection and the *n*-th purge process [96]. Then, from Equation (14) with Equation (19), the general differential equation that governs the concentration of the analyte in the coating film in time can be solved. By substituting the derived equation into the three-parameter solid model in Equation (16), the general differential equation that governs the stress–strain relationship of viscoelastic behavior can be solved, resulting that the recurrence relation between at the *n*-th and the (*n* + 1)-th purge processes and the relation between the *n*-th purge and the (*n* + 1)-th injection are found. Then, the recurrence formula can be solved, and hence the stresses at the *n*-th injection and purge processes are derived as [96]

(20) $$\sigma \left(t\right)=\left\{\begin{array}{cc} 0, & t<t_{0}\\ -\sigma_{\mathrm{sat}.}+\sigma_{\mathrm{sat}.}\alpha \overset{2\left(n-1\right)}{\underset{i=0}{\sum }}{\left(-1\right)}^{i}e^{-\frac{t-t_{i}}{\tau_{s}}}+\sigma_{\mathrm{sat}.}\left(1-\alpha \right)\overset{2\left(n-1\right)}{\underset{i=0}{\sum }}{\left(-1\right)}^{i}e^{-\frac{t-t_{i}}{\tau_{r}}}, & t_{2\left(n-1\right)}\leq t<t_{2n-1}\\ \sigma_{\mathrm{sat}.}\alpha \overset{2n-1}{\underset{i=0}{\sum }}{\left(-1\right)}^{i}e^{-\frac{t-t_{i}}{\tau_{s}}}+\sigma_{\mathrm{sat}.}\left(1-\alpha \right)\overset{2n-1}{\underset{i=0}{\sum }}{\left(-1\right)}^{i}e^{-\frac{t-t_{i}}{\tau_{r}}}, & t_{2n-1}\leq t<t_{2n} \end{array}\right.,$$

with

(21) $$\sigma_{\mathrm{sat}.}=\frac{1}{3}E_{R}K_{p}C_{g}v_{a},\;\alpha =\frac{1}{\tau_{s}}\left(\frac{E_{U}}{E_{R}}-\frac{\tau_{s}}{\tau_{r}}\right){\left(\frac{1}{\tau_{s}}-\frac{1}{\tau_{r}}\right)}^{-1}.$$

The analytical solution in Equation (20) based on the derived model shows good agreement with the trends observed in the experimental results measured by MSS coated with different viscoelastic materials, including polymers and inorganic nanoparticles [96]. Furthermore, as described above, the analytical solution based on Wenzel’s model does not predict an entire shape of a signal response when the measured signal responses do not reach a steady state (Figure 7a). Conversely, in the case of the multistep injection purge model in Equation (20), the curves predicted by the extracted parameters fit well with the experimental results, as shown in Figure 5b.

As described above, the understanding of the interactions between a receptor layer and an analyte provides a guideline to design and develop an effective receptor material. The models based on the sorption-induced nanomechanical sensing can effectively extract the indices related to the viscoelastic properties of the receptor materials as well as the interactions with the analytes. Moreover, the analysis of the transient response using the above-derived models will be beneficial for the improvement in the recognition accuracy of target analytes based on scientific interpretation. The optimized parameters can be extracted based on the sorption kinetics, and the optimized parameters can be directly used as effective indices for the identification of gas species, as proposed by Imamura et al. [99,100]. Therefore, the analytical solutions derived above can be utilized for the analyses of the static mode nanomechanical sensing signals, contributing to the development of practical artificial olfaction.

### 2.4. Membrane-Type Surface Stress Sensor (MSS)

**Figure 8:**
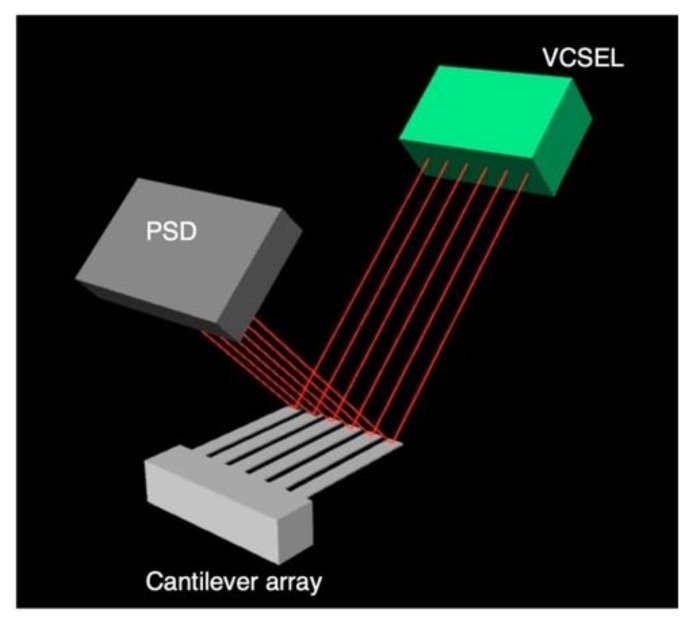
A typical setup for the optical (laser) readout system. VCSEL is usually used as a source of multiple laser light. Each laser light reflected on the surface of each cantilever is measured by PSD.

A microcantilever is the most fundamental geometry of nanomechanical sensors. Most studies employ optical laser-based detection of the cantilever deflection in both modes [36,40,41,45,46,101,102,103,104,105,106,107]. In this optical readout system, laser light emitted from, e.g., a vertical cavity surface emitting laser (VCSEL) and reflected on the surface of cantilevers is measured by a position-sensitive detector (PSD) (Figure 8). This optical readout, however, causes several practical problems for actual applications, including artificial olfaction, e.g., a bulky laser system, time-consuming laser alignment, and less applicability for large one- or two-dimensional arrays. 

To overcome these problems, the electrical readout of cantilever-type sensors has been investigated. One of the promising solutions is the use of lever-integrated piezoresistive sensing [108,109,110,111,112,113,114,115,116,117,118,119]. Several studies have been reported to improve the sensitivity of piezoresistive cantilever-type sensors for surface stress sensing applications by structural modification [57], such as making a through hole [120], the patterning of a cantilever surface [121], or the variation of geometrical parameters (e.g., length, width, and overall shapes) [122,123,124]. All these approaches rely on suppressing one of the isotropic stress components, and thus have yet to yield stress large enough to make piezoresistive detection comparable to the optical readout approach.

To realize the appropriate scheme for the enhancement of sensitivity, it is important to note the basic properties of a piezoresistive cantilever-type nanomechanical sensor for surface stress sensing, i.e., piezocoefficient [38]. Because of its high piezocoefficient, *p*-type piezoresistors created by boron diffusion onto a single crystal silicon with (100) surface can be effectively utilized [125,126,127]. Assuming plain stress (i.e., $\sigma_{z}=$ 0) owing to the intrinsically two-dimensional feature of surface stress, the relative resistance change can be described as follows [127,128]:

(22) $$\frac{\Delta R}{R}\approx \frac{1}{2}\pi_{44}\left(\sigma_{x}-\sigma_{y}\right),$$

where $\pi_{44}$ (ca. 138.1 × 10^–11^ Pa^−1^) is one of the fundamental piezoresistance coefficients of the silicon crystal; $\sigma_{x}$, $\sigma_{y}$, and $\sigma_{z}$ are stresses induced on the piezoresistor in [110], [1–10], and [001] directions of the crystal, respectively. Note that the positive/negative signs of $\sigma_{x}$ and $\sigma_{y}$ are related to the longitudinal/transversal piezoresistive effect in the <110> crystal directions of *p*-type (001) silicon. According to Equation (22), both enhancement of $\sigma_{x}$ ($\sigma_{y}$) and suppression of $\sigma_{y}$ ($\sigma_{x}$) are required to yield a substantial amount of ΔR/R. In the case of surface stress sensing, however, the stress is basically isotropic, that is, $\sigma_{x}$ is almost equal to $\sigma_{y}$, resulting in ΔR/R≈0. Therefore, the resultant signal is virtually zero on the whole surface. Because of this intrinsic material property, it is difficult to significantly improve sensitivity as long as simple cantilever-type structures are considered.

**Figure 9:**
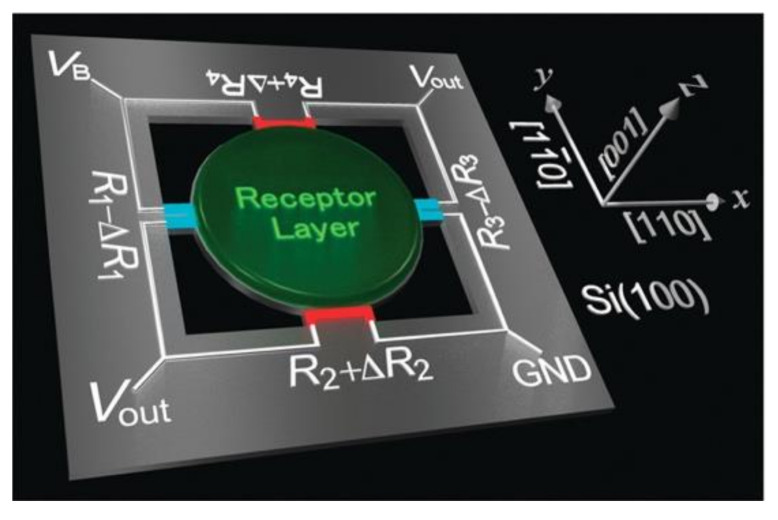
Schematic illustration of MSS with *p*-type piezoresistors on *n*-type single crystal Si(100). In this configuration with current flowing in *x*-direction, blue ($R_{1}$ and $R_{3}$) and red ($R_{2}$ and $R_{4}$) colored piezoresistors give opposite signs in $\Delta R_{i}/R_{i}$ in response to the surface stress induced on the adsorbate membrane. Reprinted with permission from Ref. [38]. Copyright 2012, American Chemical Society.

Taking account of this intrinsic problem, Yoshikawa et al. have comprehensively analyzed strain amplification schemes for sensing applications based on the strategies of the constriction and double lever geometries, leading to a development of a specific geometry: MSS [38,39]. Figure 9 shows the basic configuration of the MSS consisting of an adsorbate membrane supported with four sensing beams, in which piezoresistors are embedded, comprising a full Wheatstone bridge. The membrane is coated with a receptor layer, which generates the surface stress induced by mechanical deformation. The surface stress on the membrane is transduced to the four sensing beams as amplified uniaxial stress, resulting in the changes in the electrical resistance of the piezoresistors [38,39]. The signal output of MSS ($V_{\mathrm{out}}$) is provided by the change in total output resistance obtained from the built-in Wheatstone bridge circuit expressed as [38,39]

(23) $$V_{\mathrm{out}}=\frac{V_{B}}{4}\left(\frac{\Delta R_{1}}{R_{1}}-\frac{\Delta R_{2}}{R_{2}}+\frac{\Delta R_{3}}{R_{3}}-\frac{\Delta R_{4}}{R_{4}}\right),$$

where $V_{B}$ is the bridge voltage applied to the Wheatstone bridge circuit and $\Delta R_{i}/R_{i}$ is the relative resistance change in each sensing beam (Figure 9). In the case of MSS structure, the dominant stresses induced on the membrane are $\sigma_{x}$ for $R_{1}$ and $R_{3}$ and $\sigma_{y}$ for $R_{2}$ and $R_{4}$, resulting in opposite signals for the relative resistance changes in each set of resistors (see Equation (22)) [39]. Therefore, the entire induced surface stress can be efficiently utilized, resulting in high sensitivity, a self-compensated low-drift operation with a full Wheatstone bridge, and a stable and robust operation without a free end.

On the basis of MSS geometry, other types of MSS have been reported. For example, Seena and her group have reported the MSS deposited with indium tin oxide (ITO) [129], which have been reported to exhibit strong piezoresitance behavior [130,131]. They also applied the ITO piezoresistors further to the polymer-based MSS [132]. For another example, Yen and Chiu have designed an MSS with square holes in the membrane for the structure releasing process and achieved two-fold higher sensitivity than that of cantilever-type nanomechanical sensors [133].

## 3. Design of Receptor Materials for Nanomechanical Sensors

One of the great advantages of using nanomechanical sensors, including MSS, is the utility of wide varieties of receptor materials [55,57,58]. Unlike other types of chemical sensors, nanomechanical sensors can obtain signal responses by mechanical deformation of receptor materials derived from the sorption of target analytes. Since it has been observed that almost all kinds of solid materials, including polymers, metals, and nanomaterials, exhibit the mechanical deformation upon gas sorption, large varieties of solid materials can be utilized as a receptor material to achieve a wide range of chemical selectivity and sensitivity [55,57,58]. The variety of the receptor materials used for nanomechanical sensors in both static and dynamic mode operations are listed in Table 1. In this section, we will review some of the effective receptor designs for nanomechanical sensors, especially for MSS [134,135,136,137].One type of the receptor materials is a bulk metal. The original applications of nanomechanical sensors reported by Gimzewski et al. [40] and Thundat et al. [41] in 1994 used bulk metal film coatings as receptor layers. Gimzewski et al. used a 40 nm-thick Pt polycrystalline layer coated by vacuum deposition to monitor the catalytic reaction of hydrogen and oxygen to form water over a Pt surface in the static mode operation of cantilever-type nanomechanical sensors [40]. Thundat et al. reported the use of a 40 nm-thick Au film coated for detecting mercury vapor in the dynamic mode operation of cantilever-type nanomechanical sensors [41]. Recently, Yakabe et al. reported the effective detection of hydrogen using a 20 nm-thick Pd film coated on MSS [20,21]. They clearly demonstrated that the Pd-coated MSS detects hydrogen concentrations ranging from 5 to 40,000 ppm in a nitrogen mixture. On the basis of their sorption kinetic investigation [20,21], hydrogen molecules are dissociated on the Pd surface, and then each atom of hydrogen penetrates the bulk Pd film, resulting in the expansion of the bulk metal coating film.

**Figure 10:**
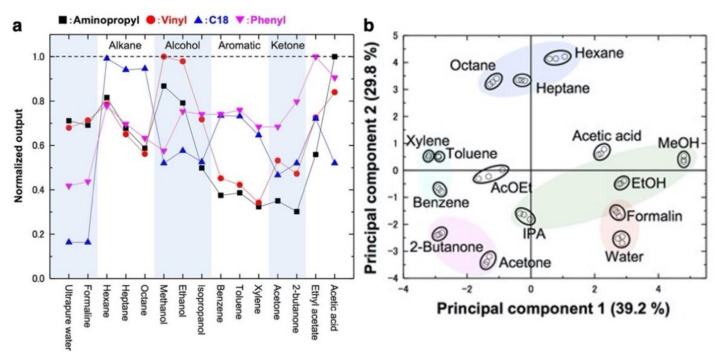
Wide varieties of chemical selectivity obtained from the surface functionalized silica-titania hybrid nanoparticles (STNPs). (**a**) Affinity trend of four different types of STNPs. (**b**) PCA scatter plot of 15 different chemicals shown in (**a**) by using the extracted features from four STNPs bearing different surface functionalities. Reprinted from Ref. [8], the authors licensed under CC-BY 4.0.

Another important material as a receptor layer of nanomechanical sensors is inorganic nanoparticles. Recent advances in nanotechnology have made it possible to synthesize a wide variety of nanoparticles with controlled sizes, shapes, and compositions. Compared to the bulk metal coating described above, inorganic nanoparticles have unique properties, such as surface functionalities and a high surface area, resulting in high chemical selectivity and sensitivity. For example, Shiba et al. developed a multistep nucleation-controlled growth method for synthesizing silica-titania hybrid nanoparticles (STNPs) with various surface functionalities including aliphatic, aromatic, and hydrophilic groups [138]. Various STNPs were coated onto each channel of MSS membranes and then the resulting MSS chip was exposed to various types of chemical analytes [8]. They obtained the different response trends by tuning the surface functionalities of the nanoparticles (Figure 10a). It was also demonstrated that the target chemical analytes can be clearly discriminated with MSS coated with the nanoparticles having different surface functionalities as presented in principal component analysis (PCA) (Figure 10b).

**Figure 11:**
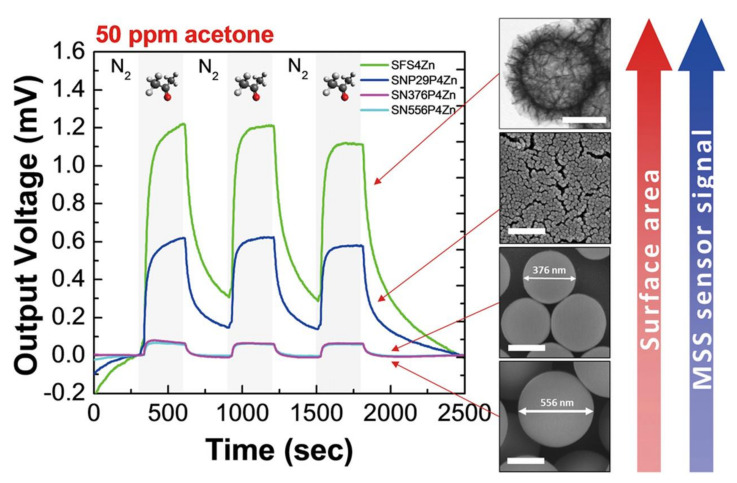
Signal response of MSS coated with Zn porphyrin-functionalized solid silica particles with diameters of 29 nm (SNP29P4Zn; blue line), 376 nm (SNP376P4Zn; light blue), and 556 nm (SNP556P4Zn; pink), or with Silica Flake–Shell bearing Zn porphyrin (SFS4Zn; green line) to 50 ppm acetone in nitrogen. Corresponding SEM micrographs are shown; the white scale bars are 250 nm. Reprinted with permission from Ref. [139]. Copyright 2017, American Chemical Society.

In contrast to the surface functionality of the nanoparticles, surface area is also an important feature of nanoparticles. By changing the sizes as well as the shapes of nanoparticles, it is possible to tune their surface area. Osica et al. synthesized silica nanoparticles whose diameters are 29, 376, and 556 nm, and coated them on MSS membranes [139]. The resulting silica nanoparticles with a larger diameter give lower signal responses because of the lower surface area, while the nanoparticles with 29 nm in diameter yielded high signal responses upon exposure to 50 ppm acetone vapor (Figure 11). Interestingly, Oscia et al. also synthesized surface nanostructured silica nanoparticles [139], i.e., a so-called silica flake–shell, which is reported by Ji et al. [140,141]. Although the size of the silica flake–shell nanoparticles is similar to that of 556 nm-sized solid silica nanoparticles, the flake–shell nanoparticles-coated MSS obtains the highest signal response to 50 ppm acetone, owing to the high surface area of the flake–shell nanoparticles [139].

Although we focused on inorganic material-based receptor layers in this section, organic molecules and polymers are also frequently utilized as an effective receptor material, as summarized in Table 1, because the chemical properties can be diversely tuned by changing their chemical structures and their functionalities. To realize practical artificial olfactory sensors, it is important to design a wide variety of receptor materials that have different chemical selectivity and prepare an array of sensors coated with those materials for pattern recognition-based analyses.

## 4. Applications for Artificial Olfaction Using MSS

As we discussed above, MSS offers one of the promising platforms for practical artificial olfaction owing to its multiple advantages. Various practical applications of MSS as artificial olfaction have been reported, especially in combination with AI and machine learning approaches; for example, the ripening stages of European pears are quantitatively predicted through their odors [135,142]. In this section, we will discuss some of the practical applications of MSS as artificial olfactory sensors.

### 4.1. Smell Identification

**Figure 12:**
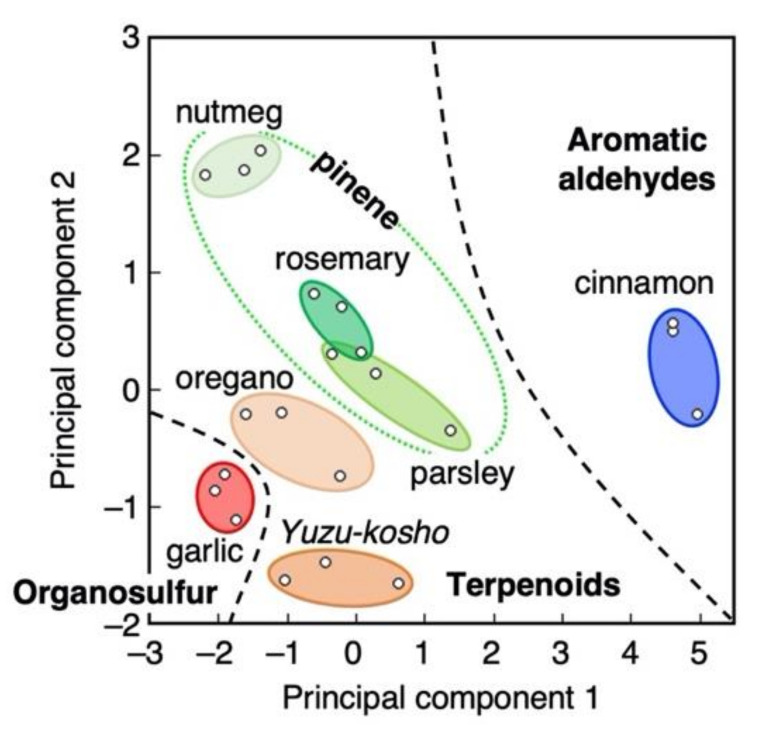
PCA scatter plot for smell identification of spices and herbs by the MSS array. The spices and herbs are categorized into three groups reflecting their major components (i.e., organosulfur, terpenoids, and aromatic aldehydes). Green dotted circle indicates the pinene-containing spices and herbs. Reprinted and modified with permission from Ref. [144]. Copyright 2016, the Japan Society of Applied Physics.

One of the key applications of artificial olfaction is to identify smells similarly to a human nose. As mentioned earlier, people identify smells through pattern recognition derived from c.a. 400 different types of olfactory receptors (Figure 1). By using this pattern recognition analysis with an array of MSS, various target analytes can be identified [8,143]. As an example of a practical application, Imamura et al. demonstrated the identification of spices and herbs [144]. They used commercially available polymers, i.e., PMMA, PAH, and PVP (see also Table 1) as receptor layers of MSS and the resulting MSS array was exposed to various vapors of spices and herbs (i.e., cinnamon, parsley, nutmeg, peppers, *Yuzukosho* (citrus-flavored pepper; one of the Japanese spices), oregano, garlic, and rosemary). By applying PCA, they distinguished each spice and herb as well as classified them into three major groups related to their major components, such as their terpenes and terpenoids, organosulfurs, and aromatic aldehydes (Figure 12) [144]. 

**Figure 13:**
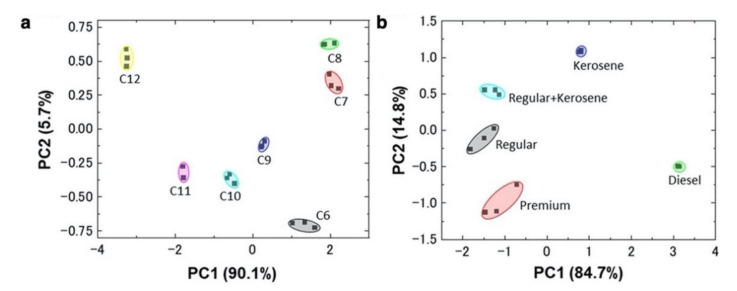
Identification of fuel oils by MSS through PCA. (**a**) PCA scatter plot of the identification of linear aliphatic hydrocarbons. (**b**) PCA scatter plot of the identification of fuel oils through their vapors. Reprinted from [145], the authors licensed under CC-BY-NC-ND 4.0.

Recently, Shiba et al. also demonstrated the identification of fuel oils including gasoline contaminated with kerosene [145]. Fuel oils such as gasoline, kerosene, and diesel are composed mainly of aliphatic hydrocarbons and have similar chemical properties. Furthermore, the illegal adulteration of fuel oils with a certain amount of impurities to increase their volume has become a frequent and serious problem worldwide [146,147]. They not only distinguished aliphatic hydrocarbons with different chain length but also differentiated the fuel oils, such as regular gasoline, premium gasoline, kerosene, and diesel with 10% kerosene-contaminated gasoline by PCA (Figure 13) [145]. The demonstration of such practical discrimination performance in a sensing device with practical specifications such as palm-top size, room temperature operation, a low power consumption, mechanical and electrical stability, and a quick response is expected to serve as a sensing platform to solve the serious problem of the fuel adulteration in the real environments.

**Figure 14:**
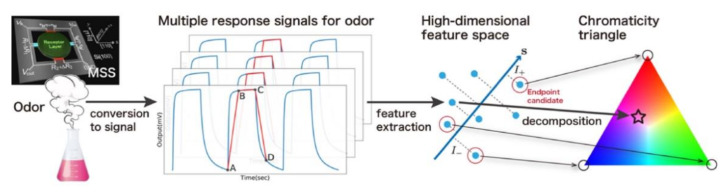
Overview of the determination of quasi-primary odor by combining machine learning and nanomechanical sensing. In the first step, the odor samples are converted to response signals with the MSS. From MSS signal responses, characteristic features are extracted. By performing machine learning-based endpoint detection, selected numbers of quasi-primary odors are determined (in this case, three). They are placed at the vertices on a chromaticity triangle and the other odors are expressed as a mixture ratio of the three quasi-primary odors, resulting in the color representation of each sample. Reprinted from Ref. [10], the authors licensed under CC-BY 4.0.

**Figure 15:**
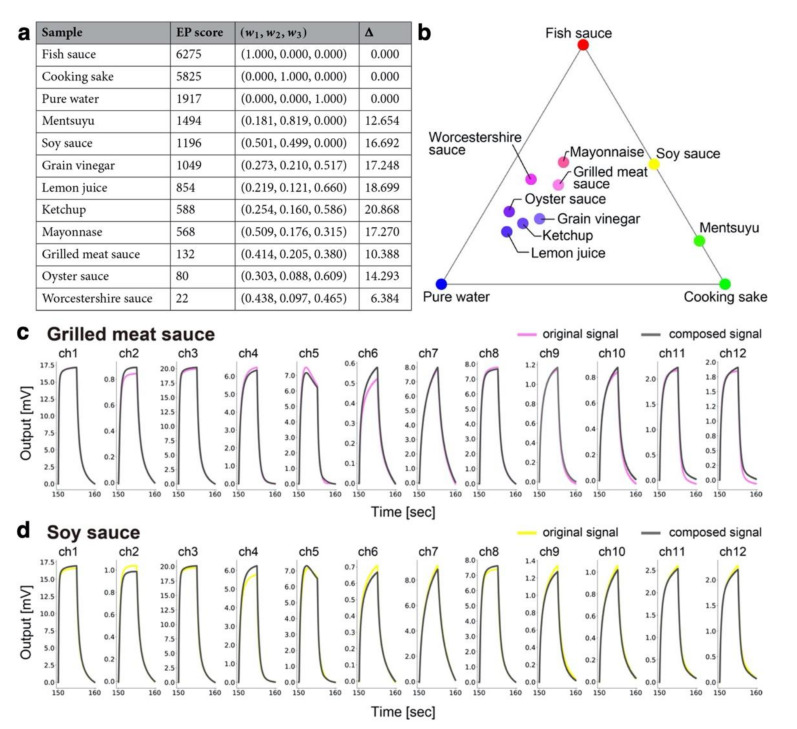
Determination of quasi-primary odors by endpoint detection. (**a**) Endpoint (EP) scores, mixture ratio ($w_{1}$, $w_{2}$, $w_{3}$), and difference Δ between original and composed signals. (**b**) Color map of pure water and 11 seasonings. Fish sauce (red), cooking sake (green), and pure water (blue) are selected as quasi-primary odors. (**c**,**d**) Original MSS signals and the composed signals for grilled meat sauce (**c**) and soy sauce (**d**). Reprinted from Ref. [10], the author licensed under CC-BY 4.0.

As mentioned earlier, pattern recognition analysis using nanomechanical sensors can classify odors of spices and herbs based on their major components. However, unlike sight (primary colors known as red, green, and blue) or taste (primary tastes known as sweet, sour, salty, bitter, and umami) composed of a rather limited number of receptors, the determination of primary odors is still challenging because there are ca. 400 different olfactory receptors with their complex cross selectivity among them [34,148]. Therefore, scientists have still been exploring and trying to alternatively define primary odors [148,149]. Recently, Xu et al. proposed an effective approach for determining “quasi” primary odors in combination with an MSS array and machine learning (Figure 14) [10]. They demonstrated this approach of determining “quasi-primary” odors out of a limited number of odor samples rather than determining true primary odors out of a full range of odors. As an example, 12 liquid samples (pure water and 11 different seasonings) were analyzed and three odor sources (fish sauce, cooking sake, and pure water) were selected as quasi-primary odors by endpoint detection (Figure 15) [10]. The flow of endpoint detection is as follows. First, from the signal responses of MSS to each odor sample, the d-dimensional features of the *i*-th odor sample are extracted as xi∈Red. The feature matrix X is defined as $X=\left(x_{1},\;x_{2},\;\ldots ,\;x_{N}\right)$ when the number of samples is N. This matrix is standardized so that a mean and a variance of each column are zero and one, respectively. Next, they set K as a large real number and generate K random d-dimensional unit vectors $\left\{s_{k}\right\}\;\left(k=1,\ldots ,K\right)$. All data X are projected onto $s_{k}$, i.e., calculate $y=X^{T}s_{k}$, which are the coordinates of each sample in the $s_{k}$ direction. Then, the indices of the maximum and minimum values of y are obtained as $I_{-}={\arg\;\min}_{i}\;y$ and $I_{+}=\arg\;\max_{i}\;y$, respectively. These samples are considered as the endpoint in the $s_{k}$ direction. Thus, the endpoint ranking can be estimated by counting the number of times (endpoint scores) when each sample is considered as an endpoint in various directions, i.e., $\left\{s_{k}\right\}\;\left(k=1,\ldots ,K\right)$. This ranking for 12 liquid samples is shown in Figure 15a.

As the quasi-primary odors are determined, all the other seasonings can be quantified in terms of the mixture ratios of these quasi-primary odors as a linear combination by performing quadratic programming (Figure 15) [10]. In addition, using the obtained mixture ratio, each odor sample can be expressed by “color” when the primary colors (red, green, and blue) are assigned to of the quasi-primary odors. Accordingly, the color map of seasonings is obtained as Figure 15b. To find “real” primary odors, it is required to examine all the combinations of odors and designated sensors, which is unrealistic. In contrast to the “real” primary odors, the proposed approach can determine a certain number of quasi-primary odors and represent any other odor in a given dataset. Thus, this approach may possess various applications to decompose, synthesize, and visualize smells in the wide range of fields, including food and cosmetic fields, where people are usually interested in a certain set of odors rather than comparing with random odors.

### 4.2. Quantification of Gas Components

**Figure 16:**
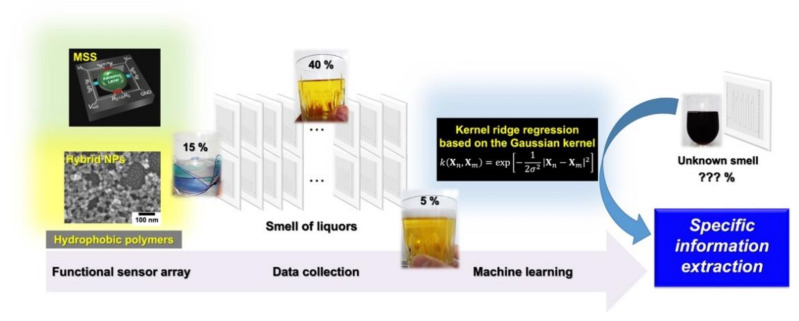
Overview of the quantification of alcohol content from the smell of liquors by combining machine learning and nanomechanical sensing. In the first step, the odor sample is converted to response signals with the MSS. From the signals, characteristic features are extracted. By performing machine learning-based regression, alcohol content is determined. Reprinted from Ref. [8], the author licensed under CC-BY 4.0.

**Figure 17:**
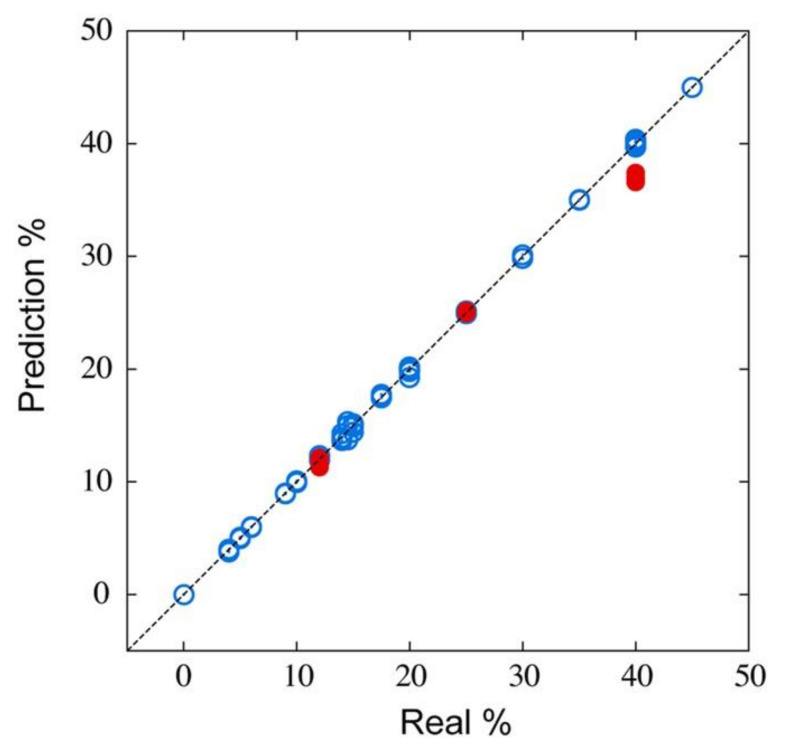
Parity plot of predicted alcohol content versus real alcohol content under an ambient condition. The blue open circles represent the known liquors which are used to train the machine learning model. The red solid circles are the unknown liquors: red wine (12%), *Imo-shochu* (25%), and whisky (40%). Reprinted from Ref. [8], the author licensed under CC-BY 4.0.

One of the representative applications of artificial olfaction is the quantification of a target analyte in a certain smell composed of a complex mixture of gaseous molecules. A chromatographic approach is a straightforward way to quantitatively analyze smells; however, it is time consuming and requires trained operators. A pattern recognition approach is useful for distinguishing a sample from others, as described above. In addition to the discrimination, the possibilities of the quantification through the pattern recognition analysis were investigated on the basis of the multiple sensor responses [150], whereas it has been experimentally demonstrated that it is practically impossible to extract specific values directly and quantitatively from a complex sample with three or more components by a conventional approach. Shiba et al. demonstrated that an array of MSS combined with a machine learning regression technique can derive quantitative information from the smells; in this case, alcohol contents in various liquors (Figure 16) as an example [8]. The surfaces of MSS membranes were coated with four different receptor materials and 35 liquid samples, including water, teas, liquors, and water/ethanol mixtures, were exposed to the MSS array to obtain the signal responses. The MSS signals were then processed by using kernel ridge regression. From the prediction accuracies of machine learning, it is found that the hydrophobic receptor materials are useful for the prediction of an alcohol content. Using four types of hydrophobic materials, they succeeded in quantifying the alcohol contents of not only known liquors, which is used for constructing a machine learning model as a training dataset (represented as blue open circles in Figure 17), but also unknown liquors including red wine, *Imo-shochu* (spirits distilled from sweet potatoes), and whisky, that were not used to train the prediction model (represented as red solid circles in Figure 17). Very recently, the Asahi Kasei Corporation in collaboration with Japanese Sake Brewery, Yoshinogawa Co., Ltd. has demonstrated the quantification of alcohol content through the odors of Japanese sake during the sake brewing process [151] and is accelerating its efforts toward a social implementation of MSS-based artificial olfaction. Moreover, Shiba et al. also reported another quantitative analysis using MSS based on the machine learning approach [9]. They measured vapors of a ternary mixture consisting of water, methanol, and ethanol. From the obtained MSS signals, they constructed prediction models based on Gaussian process regression, resulting in the successful estimation of the concentration of each component. In addition, this research was started with six types of surface functionality-bearing nanoparticles as receptor materials. From the machine learning results, it was found that receptors with a mixture of alkyl (C_18_) and amine (NH_2_) functionalities are important, and by developing new nanoparticles with varying the mixture of these functionalities, high prediction accuracy was achieved. In other words, data-driven analysis has provided a guideline for material development. This ternary mixture is one of the model systems that simulate a practical situation; a target is coexisting with a structurally similar species (i.e., methanol and ethanol) under humidified conditions. These successful quantification approaches are potentially applied to deriving a variety of information from any complicated samples, and hence can be adopted as a quantitative odor analysis method in a practical situation in various fields, such as food, agriculture, cosmetics, environment, healthcare, and medicine.

### 4.3. Exhaled Breath Diagnosis

One of the most advanced and challenging applications of olfactory sensors is the monitoring of health conditions and medical diagnosis. Health check and medical diagnosis by analyzing exhaled breath samples are based on the physiological phenomenon of gas exchange occurring in the alveoli. Human blood contains chemicals that reflect physiological phenomena and metabolic conditions in the human body [5,6]. Volatile organic compounds (VOCs) are contained in the human exhaled breath through the lung during the respiratory process and are released out of the body [152]. Breath diagnosis is considered to be an innovative non-invasive approach, that allows the development of a user-friendly, simple, and intuitive diagnostic platform [153]. Thus, the applications of artificial olfaction based on various types of chemical sensors have been investigated [6]. With its high sensitivity and robustness, MSS would be a promising option for breath diagnosis.

**Figure 18:**
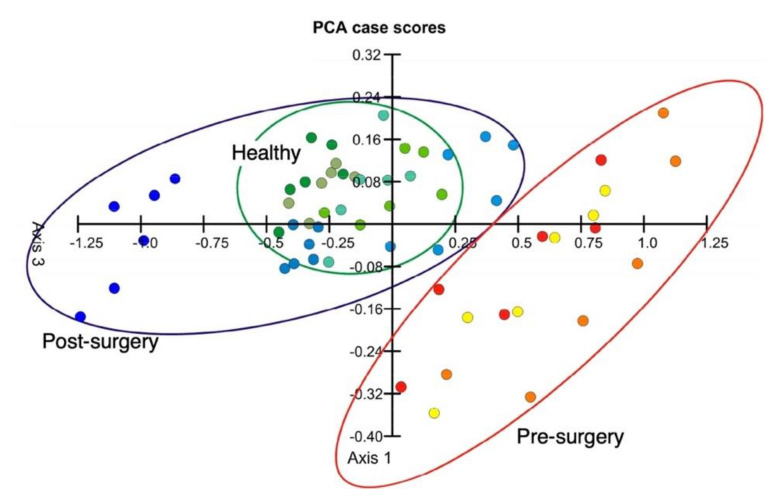
PCA scatter plot showing three distinct clusters representing healthy control persons, head and neck squamous cell carcinoma patients before and after surgery. Reprinted from Ref. [156], the author licensed under CC-BY 4.0.

Cancer is one of the most sever diseases in the world with the highest mortality. As a preliminary study, Loizeau et al. demonstrated the cancer diagnosis through breath analysis using an MSS array [154,155]. They used 16 different polymers as a receptor material, which have different chemical and physical properties to express a wide range of chemical selectivity. Breath samples collected from both healthy persons and head and neck cancer patients were analyzed in a double-blind trial. The MSS signals were analyzed by PCA. They achieved a successful discrimination of head and neck cancers from healthy persons through the analysis of the exhaled breath samples. In addition, if a cancer patient undergoes surgery to remove a tumor, the patient’s breath is expected to have a similar expiratory pattern to that of a healthy person. To demonstrate this concept, Lang et al. measured the breath samples from three head and neck squamous cell carcinoma patients before and after surgery with four healthy persons [156]. By analyzing the MSS signals by PCA, the breath samples from a post-surgery person are clearly distinguished from the breath samples from a pre-surgery person and are classified as the exhaled breath from the healthy persons (Figure 18). Although the number of samples examined in these investigations was still limited, the differentiation accuracy is expected to be improved by increasing the number of breath samples in combination with the integration of advanced machine learning algorithms.

Although the above-mentioned studies have demonstrated a certain sensitivity and specificity of MSS in breath diagnostics, it is known that the signals measured by olfactory sensors based on chemical sensor arrays including MSS are affected by the sample conditions such as temperature, humidity, and interfering gases. To evaluate the reproducibility and applicability of MSS-based olfactory sensors to the practical breath analysis, a statistical evaluation of a large number of breath samples collected over a long period of time is required. Recently, Inada et al. have conducted a statistical evaluation of total expiratory breath samples collected throughout more than a year under controlled measurement conditions [157]. They demonstrated that the key to achieving a reasonable reproducibility is to reduce the undesired effects, such as interfering exogenous gases and humidity, stemming from the differences between the sample and the purge gases. They proposed a protocol that compensates for each of the two typical inconsistencies between the sample and the purge gases by combining total expiratory breath sampling and room air purging and by reducing the contributions of the humidity in a purge gas, respectively. Following this protocol, they confirmed that a test substance in the exhaled breath can be detected. Using this protocol with the optimized MSS array, we are collaborating with Ibaraki Prefectural Central Hospital and Faculty of Medicine, University of Tsukuba to conduct studies on the exhaled breath diagnosis of cancer and are increasing the number of exhaled breath samples to improve the differentiation accuracy for the practical medical diagnosis.

### 4.4. Olfactory Sensors without Any Flow Controls

Almost all systems for the artificial olfaction and e-nose applications require devices to control gas flow, such as pumps and MFCs to obtain comparable sensing signals. Needless to say, the above-described examples used such devices. More importantly, to obtain accurate pattern recognition analysis for artificial olfaction, effective features such as slope, area, and decay time must be extracted in the same manner. However, the features strongly depend on the gas input patterns (e.g., gas flow control). Thus, the gas input patterns must be strictly fixed by using the device to control gas flow for every measurement. To resolve this intrinsic problem, data analysis methods in system identification have been developed [158]. Nakamura et al. demonstrated that the signal responses of QCM-based sensors can be analyzed by an autoregressive model [159]. They also derived a method based on a transfer function for extracting time constant from the dynamic behavior of signal responses with varying gas concentrations [160]. Furthermore, pioneering works were done by Marco, Pardo, and coworkers to adapt non-linear models, including artificial neural networks (ANN) and Wiener kernel analysis, for describing the complex response of sensing systems using QCM [161,162,163]. Most of the models well describe the change in the flow system; however, the studies still used such devices to demonstrate the models by changing the input patterns of the gas flow.

The most challenging measurement system is an open sampling condition, in which sensors are directly exposed to sample gases without any gas flow control. In this case, the gas input pattern is neither controlled nor monitored. Several research groups reported the gas identification in the open sampling conditions [164,165,166,167,168,169,170,171,172]. While gas identification protocols without any gas flow control are used in these studies on gas identification under open sampling conditions, the signal features used in the studies depend on gas input patterns, giving rise to limited measurement conditions. To realize a gas identification that is highly robust to gas input patterns, it requires analytical methods based on signal features that are intrinsically independent of the gas input patterns, that is, features that are determined solely by the combination of sensors and gas species. Thus, further breakthroughs have been demanded to improve the usability of the measurement system, such as robustness and portability, towards the practical application of artificial olfaction.

Imamura et al. recently developed a gas identification protocol under the open sampling condition using the transfer function ratio, which is intrinsic to gas species and is independent of the gas input patterns [173]. A transfer function is one of the mathematical representations to describe an input–output relationship. When a gas sensing system exhibits a linear response, in which an output sensing signal $y\left(t\right)$ is linear in the gas injection pattern $x\left(t\right)$, $y\left(t\right)$ can be obtained as a convolution of $x\left(t\right)$ and the time-domain transfer function (of the impulse response function) $h_{g}\left(t\right)$ [173]:

(24) $$y\left(t\right)=\int_{0}^{t}h_{g}\left(\tau \right)x\left(t-\tau \right)d\tau ,$$

where t and g denote the time and gas species, respectively. As $h_{g}\left(t\right)$ is considered an intrinsic function of the gas, gas species can be identified by $h_{g}\left(t\right)$. Since $h_{g}\left(t\right)$ is independent of $x\left(t\right)$, one of the biggest advantages of the use of $h_{g}\left(t\right)$ as a feature is that measurement data obtained through different gas input patterns become comparable. By applying the Fourier transform, the frequency-domain expression for Equation (24) can be obtained as

(25) $$Y\left(f\right)=H_{g}\left(f\right)X\left(f\right),$$

where $X\left(f\right)$, $Y\left(f\right)$, and $H_{g}\left(f\right)$ are the frequency-domain expressions for the gas input, output signal, and the transfer function, respectively. Gas species can be identified by the transfer function $H_{g}\left(f\right)$ directly calculated from the gas input pattern $X\left(f\right)$ (e.g., gas flow rate or gas concentration) and sensing signal $Y\left(f\right)$; however, it is required to measure $X\left(f\right)$ by controlling and/or monitoring gas injections. To overcome this problem, they further demonstrated gas identification using an array of MSS with different sensing characteristics [173]. Considering that a gas g is introduced into a gas sensor array according to gas input $X\left(f\right)$, the output signal of the *i*-th channel of the sensor array $Y_{i}\left(f\right)$ can be written as

(26) $$Y_{i}\left(f\right)=H_{g,i}\left(f\right)X_{i}\left(f\right),$$

where $H_{g,i}\left(f\right)$ is the transfer function of the *i*-th channel for the gas *g* [173]. If each channel of the sensor array can be considered spatially equivalent to the gas input, it is possible to assume that $X_{i}\left(f\right)$ is the same for all the channels, i.e., $X_{i}\left(f\right)=X\left(f\right)$. Thus, for any combination of two channels *m* and *n*, from Equation (26), the following equation holds [173]

(27) $$X\left(f\right)=\frac{Y_{m}\left(f\right)}{H_{g,m}\left(f\right)}=\frac{Y_{n}\left(f\right)}{H_{g,n}\left(f\right)}.$$

**Figure 19:**
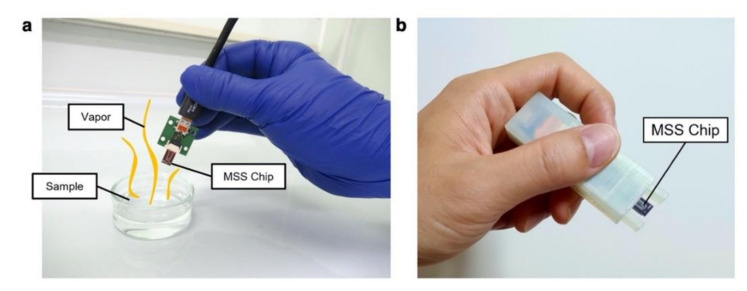
Free-hand measurement setup. Note that this approach requires only a sensor array and a readout device. (**a**) Picture of the free-hand measurement setup. (**b**) Picture of a wireless free-hand measurement device. Reprinted from [173,174], the author licensed under CC-BY 4.0.

Then, Equation (27) can be rewritten as

(28) $$K_{m,n}\left(f\right)=\frac{H_{g,m}\left(f\right)}{H_{g,n}\left(f\right)}=\frac{Y_{m}\left(f\right)}{Y_{n}\left(f\right)},$$

where $K_{m,n}\left(f\right)$ is the transfer function ratio. As can be seen in Equation (28), $K_{m,n}\left(f\right)$ can be obtained from the output signal ratio of the *m*-th and *n*-th channels of the array in the frequency domain with any gas input pattern. Therefore, it is possible to identify a gas species without controlling or monitoring the gas input pattern by calculating $K_{m,n}\left(f\right)$ from an arbitrary combination of two channels of a sensor array. They demonstrated the identification of spices and herbs through their smells (i.e., rosemary, red chili pepper, and garlic) [173]. The odors of the spices and herbs were measured with MSS coated with four different inorganic nanoparticles bearing different functional groups. By applying the transfer function ratio obtained from the ratio of the signals in Equation (28) with machine learning algorithms, they achieved the gas identification of the spices and herbs up to 89% accuracy. This novel type of gas identification protocol realizes a compact measurement system in which gas species can be identified through a *free-hand* measurement (Figure 19) [173,174].

## 5. Conclusions and Perspective

Nanomechanical sensors are attracting more and more attention as a potential platform for the artificial olfaction in combination with data processing technologies, including machine learning techniques. The theories briefly described in this review have promoted the advances and developments in the nanomechanical sensing systems, allowing for interpretations and understandings of the experimental results. We summarized a wide variety of coating films that can be used for nanomechanical sensors. Those receptor materials have helped realize various practical applications of nanomechanical sensors for the artificial olfaction. In addition, machine learning approaches have enabled nanomechanical sensors to make significant advances as olfactory sensors, achieving the quantification of target components and the determination of quasi primary odors. Thereby, nanomechanical sensors have proven their applicability to artificial olfaction and are expected to contribute to many fields.

In this review, we featured a specific geometry of a nanomechanical sensor, MSS, which possesses multiple advantages for practical applications including high sensitivity, compactness, room temperature operation, mechanical/electrical stability, low power consumption, and quick response. Although MSS can be utilized for various applications as olfactory sensors, practical artificial olfaction devices are not ready yet as several challenges still need to be overcome, such as the development and optimization of the receptor materials to improve chemical sensitivity and selectivity, precise calibration of signals with standard gases, mass production of sensor chips and devices, efficient connections with edge computing and cloud systems, and so forth. These challenges require the integration of science and technologies through a collaboration with academics and the industry. To integrate all the required cutting-edge technologies, MSS Alliance, an industry–academia–government joint research framework, was launched in 2015 by the National Institute for Materials Science (NIMS), Kyocera Corporation, Osaka University, NEC Corporation, Sumitomo Seika Chemicals Co., Ltd., Asahi Kasei Corporation (from April 2017), and NanoWorld AG. [175,176]. Each member of the MSS Alliance, with their own expertise, contributed to the development of various technologies, which are required for practical artificial olfaction. To encourage interested companies and research institutes to conduct demonstration experiments, the MSS Forum was launched in 2017 [142,177]. From 2020, the MSS Forum has been an open platform for sharing the latest information on MSS, artificial olfaction, and its social implementation. To effectively integrate the state-of-the-art technologies accumulated through the MSS Alliance and MSS Forum towards the social implementation of MSS-based olfactory sensors, we are developing related science and technologies. We hope that these efforts will eventually lead to the actual contributions of olfactory sensors in various fields, including the food, environment, agriculture, healthcare, and medicine fields.

**Table 1:** List of receptor materials used for nanomechanical sensors including cantilever-type sensors and MSS in both static and dynamic mode operations.

Receptor Materials	MSS (Static)	Cantilever (Static)	Cantilever (Dynamic)
*Small molecules*			
Calix[*n*]arene	—	[178]	—
Cu complex	[179]	—	—
Cyclodextrin	—	[178]	—
Metallo-phthalocyanine	—	[178]	—
Metallo-porphyrins	[139,180]	—	—
Porphyrins	[139,180]	—	—
Squalene	—	[178]	
*Polymers* ^1^			
CAB	[42,154]	—	—
CMC	[154,156]	—	[181]
Dextran	[154,182]	[182]	—
Gelatin	—	—	[183,184]
HPC	[156]	—	—
Tenax	[157]	—	—
P4MS	[96,157,173,185]	—	—
PAA-AA	[156]	—	—
PAH	[144,145]	[118]	[181]
PCL	[8,96,173,185,186]	—	—
PDPP	—	[178]	—
PECh	[174]	[178]	—
PEG/PEO	[99,156]	[187]	[181]
PEG-MEMA	[156]	—	—
PEI	[38,156]	[118]	[181]
PEMA	—	[188]	—
PHEMA	—	[189]	[189]
PIB	[156]	[178]	—
PLL	—	[190]	—
PMMA	[10,92,99,144,145]	[35,36,37,188,191]	[36,157,174,181,192]
PS	[37,96,185,186]	[189]	[189]
PS-AA	[174]	—	—
PSS	[39,89,154]	—	[181]
PSU	[8,173]	—	—
PU	—	[37]	—
PVA	—	[118]	[181]
PVC	[99]	—	—
PVF	[173,185]	—	—
PVP	[89,144,193]	[118]	—
PVPh	[174]	—	—
PVPy	[154,156]	[37]	[181]
*Inorganic nanomaterials*			
Copper nanorods	—	—	[194]
Gold NPs	[195]	[196]	[197,198,199]
Silica NPs	[9,10,139,173]	—	[200,201]
Nanostructured silica NPs	[139,202]	—	—
Silica-Titania hybrid NPs	[8,9,10,96,143,145,173,203]	—	—
TiO_2_@MnO_2_ nanorods	—	—	[204]
ZnO@Si nanorods	—	—	[205,206]
*2D materials*			
Graphenes	[207,208,209]	[190,210]	[43,197,198]
MoS_2_	[208]	—	—
WS_2_	[208]	—	—
*Self-assembled monolayer*			
Alkanethiols	—	[45,181,211]	[212,213,214]
Carboxylated thiols	[133]	[35,36,181,215,216]	—
Aminated silane	—	[217]	[206]
DNA	—	[46,47,48,102,117,218,219]	[220]
Proteins	—	[103,133,215,216,221,222,223,224,225,226]	[223]
*Metal films*			
Au	—	[35,36]	[41,44,63]
Cr	—	[227]	—
SiN	—	—	[192]
Pd	[20]	—	—
PdCuSi	[21]	—	—
Pt	—	[36,40]	—
*Other materials*			
Carbon nanotubes	—	[190]	—
MOFs ^2^	[228]	—	[229]
Zeolites	[193]	—	—

^1^ Abbreviations for polymers: CAB, cellulose acetate butyrate; CMC, carboxymethylcellulose; HPC, hydroxypropyl cellulose; P4MS, poly(4-methylstyrene); PAA, poly(acrylic acid); PAA-AA, poly(acrylic acid)-acetic acid; PAH, poly(allylamine hydrochloride); PCL, polycaprolactone; PDPP, poly(diphenoxyphosphazene); PECh, polyepichlorohydrin; PEG, poly(ethylene glycol); PEG-MEMA, poly(ethylene glycol methyl ether)-methylmethacrylate; PEI, poly(ethylene imine); PEMA, poly(ethyl methacrylate); PEO, poly(ethylene oxide); PHEMA, poly(hydroxy ethyl methacrylate); PIB, polyisobutylene (butyl rubber); PLL, poly-L-lysine; PMMA, poly(methylmethacrylate); PS, polystyrene; PS-AA, poly(styrene-co-allyl alcohol); PSS, poly(sodium 4-styrene sulfonate); PU, polyurethane; PVA, poly(vinyl alcohol); PVC, poly(vinyl chloride); PVF, poly(vinylidene fluoride); PVP, poly(vinyl pyrrolidone); PVPh, poly(4-vinylphenol); PVPy, poly(vinylpyridine). ^2^ Metal–Organic Frameworks.

## Data Availability

The data presented in this review are available from the corresponding author upon reasonable request.
